# Insecticide Resistance and Vector Control

**DOI:** 10.3390/tropicalmed11010033

**Published:** 2026-01-22

**Authors:** Adriana E. Flores, Jesus A. Davila-Barboza, Alan E. Juache-Villagrana

**Affiliations:** 1 Facultad de Ciencias Biológicas, Universidad Autónoma de Nuevo León (UANL), San Nicolás de los Garza 66455, NL, Mexico; jdavilab@uanl.edu.mx; 2 Avia-GIS, Risschotlei 33, 2980 Zoersel, Belgium

Insecticide-based strategies have been central to vector control programs targeting diseases of human and veterinary importance for decades. While these interventions have substantially reduced disease transmission, their sustained, often intensive, use has driven the widespread emergence of insecticide resistance. This Special Issue, “Insecticide Resistance and Vector Control”, brings together contributions that reflect the historical roots, current challenges, and emerging directions of resistance research across diverse vector systems.

The studies included in this Special Issue highlight that insecticide resistance is not a recent or isolated phenomenon but rather the cumulative outcome of long-term chemical pressure applied across diverse ecological and operational contexts. Historical analyses of vector control programs provide an essential perspective on how patterns of insecticide use have influenced resistance trajectories, emphasizing the importance of incorporating past experience into contemporary resistance management strategies.

Advances at the molecular and physiological levels are also a central theme of this collection. Transcriptomic studies in major mosquito vectors demonstrate that insecticide exposure can trigger broad changes in gene expression, extending beyond classical detoxification pathways to include immune-related and cuticle-associated processes. These findings reinforce the view that resistance is a multifactorial trait, involving complex biological responses that may influence vector fitness, adaptation, and control outcomes.

Field-based investigations highlight the importance of evaluating resistance within realistic transmission settings. By examining vector feeding preferences, infectivity rates, and the distribution of resistance-associated mutations in natural populations, these studies illustrate how behavioral and ecological factors interact with resistance mechanisms. Such integrated approaches are critical for interpreting resistance data in ways that are relevant to operational vector control and public health decision-making.

This Special Issue also reflects the growing recognition that insecticide resistance is a shared challenge across human and veterinary health sectors. Contributions addressing arthropod pests of veterinary importance and exploring alternative formulations and delivery systems, including nano-based approaches, highlight ongoing efforts to improve control efficacy while potentially reducing selective pressure for resistance. These studies align with the broader need for innovation in vector and ectoparasite control tools as conventional compounds lose effectiveness.

Collectively, the articles in this Special Issue emphasize several key points. First, resistance should be understood as a dynamic process shaped by historical insecticide use, biological complexity, and local ecological conditions. Second, molecular and omics-based insights provide powerful tools for elucidating resistance mechanisms but must be integrated with phenotypic and field data to inform practical control strategies. Finally, sustainable vector control will depend on adaptive, integrated approaches that combine chemical interventions with complementary methods under the framework of Integrated Vector Management.

In conclusion, this Special Issue offers a concise overview of the current progress and persistent challenges in insecticide resistance research. While substantial advances have been made, important gaps remain in linking mechanistic findings to long-term operational effectiveness. We hope that this collection will serve not only as a useful reference for researchers, practitioners, and policymakers, but also as a catalyst for continued interdisciplinary efforts to preserve the efficacy of vector control interventions and reduce the global burden of vector-borne diseases.

